# Editorial: Plant Single Cell Type Systems Biology

**DOI:** 10.3389/fpls.2016.00035

**Published:** 2016-02-09

**Authors:** Marc Libault, Sixue Chen

**Affiliations:** ^1^Department of Microbiology and Plant Biology, University of OklahomaNorman, OK, USA; ^2^Department of Biology, Interdisciplinary Center for Biotechnology Research, Genetics Institute, Plant Molecular and Cellular Biology Program, University of FloridaGainesville, FL, USA

**Keywords:** systems biology, molecular phenotype, omic analyses, single cell types, root hair, trichome

The molecular responses of a plant to a stress and the molecular profiles of plant organs during their development and differentiation are the reflection of the contribution from different cell types composing the plant and the organs. Hence, a major limitation to understanding plant cellular and molecular responses in different cells is the multicellular complexity of the plant or organs used to decipher them. For instance, as mentioned by Coker et al. changes in the expression levels of plant cells infected by pathogenic microbial organisms are diluted by the relative abundance of uninfected cells. This constraint has led plant biologists to select model single plant cell types such as pollen, trichomes, cotton fiber, guard cells of stomata, and various root cell types including the root hair cells, and to develop new technologies (e.g., microscopic, biochemical, and omics) to decipher their biology (Dai and Chen, 2012; Misra et al.). However, as mentioned by Schmid et al. (2015) profiling single cell types is dependent on the quantity and purity of the samples isolated as well as the use of sensitive and accurate profiling methods. The 12 articles published in this Research Topic highlight interesting methodology and biological systems applied by plant scientists to advance our knowledge in plant biology using single cell type models.

Working at the level of single cell types is motivated by the need for analyzing specific biological information in the relevant cell types, which would otherwise be missed when using tissues or organs (Dai and Chen, 2012; Misra et al.). This is especially true when working on plant-microbe interactions where only a subset of cells are infected by pathogenic or mutualistic microbes. For instance, to precisely characterize the transcriptional response of *Arabidopsis thaliana* during infection by the oomycete *Hyaloperonospora arabidopsidis* (*Hpa*), Coker et al. applied Fluorescent Activated Cell Sorting (FACS) in separation of haustoriated and non-haustoriated Arabidopsis cells for transcriptomic analysis, allowing the discovery of 139 new *Hpa*-responsive genes and characterization of the local and systemic responses of the plant cells. Similarly, working on the infection of the soybean root hair cells by rhizobium, the nitrogen-fixing symbiotic soil bacterium, Hossain et al. described the integration of the transcriptomic, proteomic, phosphoproteomic, and metabolomic datasets to generate a comprehensive network of the early stage of the nodulation process. Another strategy applied by Chen et al. to gain a better understanding of the nodulation process is to compare the transcriptomes of different rhizobium-infected plant cell types. Specifically, they looked for the *Medicago truncatula* genes controlling infection thread formation and elongation by analyzing transcriptomic data obtained from inoculated root hair cells and the infection zone of the *M. truncatula* nodule. Studying plant reproduction, another complex biological process, can also benefit from single cell type analyses. Schmid et al. detailed novel methods to analyze single cell type molecular profiles such as the female gametophyte, which is composed of antipodal, central, egg, and synergid cells. Similarly, working on the male gametophyte, Lu et al. developed a pollen culture system for isolating generative cells, sperm cells and vegetative nuclei from tomato pollen grains. Plant reproduction studies can also benefit from the utility of unique single cell type models, such as *Equisetum arvense*, an herbaceous plant characterized by its spore reproduction. Zhao et al. analyzed the cellular and proteomic profiles of *E. arvense* during spore germination, revealing the high level activities of the heterotrophic and autotrophic metabolisms.

The generation of unambiguous datasets from single cell types is an asset for generating systems biology models as demonstrated by Kwak et al. (2008), Sun et al. (2014) and Hossain et al. Single cell types are also considered attractive systems to precisely depict molecular phenotypes. As noted by Schiefelbein, access to single cell types now opens a new area to phenotype mutants: the establishment of molecular phenotypes (i.e., distinct molecular profiles between wild-type and mutants and their changes in response to environmental stresses). Such an approach is often limited by efficient methods to generate high quality single plant cell type samples and by the limited amount of material available for analyzing the molecular phenotype. Thus, technological development must continue to meet the needs of addressing questions at the single cell type level. Nucleic acid sequencing technologies associated with the use of performant bioinformatics tools are now enabling an accurate and sensitive quantification of single cell type transcriptomes and epigenomes. As an example, the analysis of previously published Arabidopsis root hair transcriptome data sets allowed the characterization of 5409 genes differentially expressed in root hairs versus non-root hair epidermal cells and the generation of a co-expression network (Li and Lan). Similarly, biochemical methods are quickly developing allowing access to single plant cell type proteome (Svozil et al.) and metabolome (Barkla and Vera-Estrella; Bartels and Svatos; Misra et al.). Specifically, Barkla and Vera-Estrella described the differential metabolome between specialized trichome cells from *Mesembryanthemum crystallinum* named epidermal bladder cells (EBC). This analysis can be expected to provide a systems level of understanding of EBC when integrated with the existing proteomic and transcriptomic data sets. Similarly, Misra et al. reviewed the most recent advances in our understanding of the guard cell metabolome. This knowledge is essential to advance our understanding of stomatal opening and closing, which have a major impact on plant transpiration, CO_2_ uptake and pathogen immunity. At the proteome level, Svozil et al. applied Meselect, an innovative methodology to isolate leaf epidermal, vascular and mesophyll cells. Using these samples, the authors established a proteome map of each cell type and revealed cell type specific processes. These types of studies are going to be revolutionized by the development of new imaging techniques. For instance, applying infrared-laser ablation electrospray ionization (LAESI) and UV-laser desorption/ionization (LDI) methods, less intrusive and spatially-resolved analyses of the metabolomes of single plant cell types are described in this ebook (Bartels and Svatos). These technological developments have greatly enhanced our capabilities in analyzing molecular components in different cells at an unprecedented scope and depth through omics for modeling and hypothesis generation. The integration of hypothesis generation and hypothesis testing in systems biology research will ultimately lead to a holistic view of cellular processes and molecular networks in plants and will create stepping stones toward molecular breeding and biotechnology for enhanced crop stress tolerance, yield and bioenergy.

## Author contributions

ML drafted the manuscript. SC edited the manuscript.

## Funding

Research on plant single cell-type regulatory networks in the Libault laboratory has been supported by NSF grants IOS-1453613, IOS-1339194, by DOE grant DE-SC0012629, and by the Oklahoma Center for the Advancement of Science and Technology PS14-025 to ML. Research on single cell-type proteomics and metabolomics in the Chen laboratory has been supported by NSF grants MCB-0818051, MCB-1158000, and MCB-1412547 to SC.

### Conflict of interest statement

The authors declare that the research was conducted in the absence of any commercial or financial relationships that could be construed as a potential conflict of interest. The reviewer BE and handling editor declared their shared affiliation, and the handling editor states that the process nevertheless met the standards of a fair and objective review.
